# Standardizing Corneal Transplantation Records Using openEHR: Case Study

**DOI:** 10.2196/48407

**Published:** 2024-09-16

**Authors:** Diana Ferreira, Cristiana Neto, Francini Hak, António Abelha, Manuel Santos, José Machado

**Affiliations:** 1 ALGORITMI Research Center Intelligent Systems Associate Laboratory (LASI) School of Engineering, University of Minho Guimarães Portugal

**Keywords:** electronic health record, EHR, corneal transplantation, keratoplasty, openEHR, data representation, data exchange, templates, archetypes, forms, standardization

## Abstract

**Background:**

Corneal transplantation, also known as keratoplasty, is a widely performed surgical procedure that aims to restore vision in patients with corneal damage. The success of corneal transplantation relies on the accurate and timely management of patient information, which can be enhanced using electronic health records (EHRs). However, conventional EHRs are often fragmented and lack standardization, leading to difficulties in information access and sharing, increased medical errors, and decreased patient safety. In the wake of these problems, there is a growing demand for standardized EHRs that can ensure the accuracy and consistency of patient data across health care organizations.

**Objective:**

This paper proposes the use of openEHR structures for standardizing corneal transplantation records. The main objective of this research was to improve the quality and interoperability of EHRs in corneal transplantation, making it easier for health care providers to capture, share, and analyze clinical information.

**Methods:**

A series of sequential steps were carried out in this study to implement standardized clinical records using openEHR specifications. These specifications furnish a methodical approach that ascertains the development of high-quality clinical records. In broad terms, the methodology followed encompasses the conduction of meetings with health care professionals and the modeling of archetypes, templates, forms, decision rules, and work plans.

**Results:**

This research resulted in a tailored solution that streamlines health care delivery and meets the needs of medical professionals involved in the corneal transplantation process while seamlessly aligning with contemporary clinical practices. The proposed solution culminated in the successful integration within a Portuguese hospital of 3 key components of openEHR specifications: forms, Decision Logic Modules, and Work Plans. A statistical analysis of data collected from May 1, 2022, to March 31, 2023, allowed for the perception of the use of the new technologies within the corneal transplantation workflow. Despite the completion rate being only 63.9% (530/830), which can be explained by external factors such as patient health and availability of donor organs, there was an overall improvement in terms of task control and follow-up of the patients’ clinical process.

**Conclusions:**

This study shows that the adoption of openEHR structures represents a significant step forward in the standardization and optimization of corneal transplantation records. It offers a detailed demonstration of how to implement openEHR specifications and highlights the different advantages of standardizing EHRs in the field of corneal transplantation. Furthermore, it serves as a valuable reference for researchers and practitioners who are interested in advancing and improving the exploitation of EHRs in health care.

## Introduction

### Background

The eye is a highly evolved and complex sensory organ possessed by a wide range of species, enabling organisms to perceive and interpret visual information from their surroundings. Vision is one of the most valuable senses for humans and plays a critical role in every facet of an individual’s life [1]. The sense of vision is the result of an intricate interaction among the eyes, the brain, and the nervous system [2].

Visual impairment occurs when a pathological condition disrupts the visual system and one or more of its associated functions [1]. Blindness is a major public health issue, particularly in low- and middle-income countries where access to health care and resources is scarce [3,4]. The loss of sight can severely impact an individual’s daily life, hindering their ability to perform routine tasks, interact with others, and preserve their independence [5]. On many occasions, blindness can also lead to social isolation, depression, and decreased quality of life [1,5].

In 2019, the World Health Organization estimated in the World Report on Vision that there were approximately 2.2 billion people worldwide with vision impairment or blindness [1]. The prevalence of visual disability is alarming and a source of growing global concern.

The apprehension surrounding blindness is rooted not only in the physical limitations it imposes but also in its social and economic consequences [3]. From an economic point of view, the loss of sight can cause reduced workforce participation, decreased productivity, and increased health expenses [5]. Consequently, the loss of income and the higher health care costs can drain governments with additional financial pressures, exacerbating poverty and slowing economic growth [1]. Hence, the impacts of blindness are far reaching, affecting not only the individual but also their families, communities, and society as a whole.

Without more assertive measures, the escalating demand for eye care services worldwide is projected to persist and intensify in the next few decades, posing a meaningful challenge to the health care industry and requiring innovative solutions to meet the increasing pressure for quality eye care services [6,7].

The eye is a complex organ composed of several structures that work together in the perception of the world in all its lights, colors, shapes, and movements [2]. One of the most vital structures of the visual system is the cornea, which is the clear outermost layer located at the front of the eye [8]. A transparent cornea acts as a clear window to allow light to enter the eye and reach the retina, a layer of neural tissue at the back of the eye where light is converted into electrical signals and transmitted to the brain for interpretation as visual information [2]. For an individual to have clear vision, the cornea must be transparent and free of any obstructions, such as scars or opacities, to allow light to cross the eye and access the retina [8,9].

The main causes of visual impairment are cataract, glaucoma, macular degeneration, detached retina, diabetic retinopathy, and retrolental fibroplasia [3,7,10,11]. Some of these eye conditions, such as cataract, diabetic retinopathy, and retrolental fibroplasia, can negatively impact the clarity of the cornea and lead to vision impairment [2,9]. According to the World Health Organization, corneal opacities are the fourth leading cause of blindness on a global scale [9].

In many cases, visual rehabilitation is possible with corneal transplantation [4,8]. Corneal blindness can be effectively reversed with a cornea transplant from a healthy donor. Because the cornea lacks blood vessels, the risk of graft rejection is significantly reduced, making corneal transplantation one of the most successful forms of organ transplantation in the human body [8]. Corneal transplantation, also known as keratoplasty, is a surgical procedure that replaces a damaged or diseased cornea with a healthy one to restore vision and improve the quality of life of patients [4].

The success of corneal transplantation heavily relies on the accurate and timely management of patient data, including preoperative evaluation, surgical planning, and postoperative care. Electronic health records (EHRs) have evolved as indispensable means for handling clinical information, providing a centralized repository for patient records, and facilitating communication between health care providers [12,13]. The appropriate use of EHRs can substantially bolster the positive outcomes of corneal transplant surgeries, culminating in improved patient results and a more seamless and efficient care journey. By minimizing errors and inconsistencies in patient data management, EHRs can further diminish the likelihood of unfavorable events, ultimately improving the success of corneal transplants.

Despite their widespread use, traditional EHRs are prone to lack of standardization and consistency [12]. As a consequence, inconsistency and fragmentation plague the management and documentation of corneal transplantation records, causing difficulties in accessing and sharing information, increased medical errors, and decreased patient safety. Furthermore, the lack of standardization and organization creates an obstacle for health care professionals to access complete and accurate information about patients who are due to undergo or have undergone corneal transplantation, resulting in inefficiencies and suboptimal patient care.

To address these issues, there is a growing need for standardized EHRs that can ensure the accuracy and consistency of patient data across health care organizations and locations [14,15] to ultimately improve the management and documentation processes of corneal transplantation records.

One promising approach for standardizing EHRs is the use of openEHR structures [16,17]. openEHR is an open-source standard that provides a set of specifications and tools to support the creation of interoperable data structures and the long-term management of health data [18]. A foundational paradigm on which the openEHR framework is based is the 2-level modeling, separating domain semantics from software. Under the model-driven approach, a stable reference information model constitutes the first level of modeling, whereas formal definitions of clinical content in the form of archetypes and templates constitute the second level. Overall, the adaptability, flexibility, and scalability of openEHR’s modular methodology provide a powerful solution to the challenges facing the health care industry, and it is an ideal approach for health care systems of all sizes [16-19].

### Objectives

Hence, this study sought to explore the implementation of openEHR structures as a means of standardizing records in the field of corneal transplantation. In addition, this manuscript expounds upon the potential benefits that may arise from the use of openEHR specifications for standardizing corneal transplantation records, including improved data consistency and completeness and increased data accessibility and sharing, alongside the mitigation of errors and inefficiencies in data management. This paper will also delve into the challenges and limitations of implementing openEHR in the context of corneal transplantation.

Through the evaluation of the potential benefits and hurdles of using openEHR specifications, this study provides an informative resource and a valuable reference for researchers and practitioners interested in improving the use of EHRs in health care, extending beyond the field of ophthalmology and encompassing other medical disciplines as well.

One of this research’s objectives was to contribute to the ongoing efforts to improve the quality and safety of patient care through the disclosure of insights into the potential of using openEHR structures for the advancement of EHRs in health care, particularly in the field of corneal transplantation.

As health care continues to evolve, the authors believe that the standardization of clinical records using openEHR structures holds great potential for ensuring that patients receive safe, effective, and high-quality care.

## Methods

### Overview

In this study, a specific methodology was defined that outlines the sequential steps involved in transforming the corneal transplantation records of a Portuguese hospital using the openEHR specifications.

In contrast to traditional approaches, openEHR’s advanced modular approach separates data from applications and services, providing a level of flexibility that is unmatched in the industry [20]. This approach enables an easier adaptation to constantly evolving requirements, technological changes, health care policies, and other external factors. Moreover, by separating the data layer from the application layer, openEHR can integrate a wide range of health-related data from various sources regardless of the format in which they are stored [20].

By using archetypes and templates as a means of ensuring the consistency and accuracy of clinical data, openEHR is more supple compared to conventional approaches to clinical documentation, which often rely on free-text entries that can be arduous to comprehend and interpret across different patients and clinical settings [20]. The use of archetypes and templates allows health care institutions to minimize variability and fragmentation, which contrasts with the current uncoordinated methods. It provides a framework that allows health care professionals to customize patient-specific information in a more dynamic and structured way [19]. In addition, it ensures that clinical data remain consistently structured and semantically interoperable, leading to more precise interpretation and sharing of clinical data among different clinical systems.

**Figure 1:**
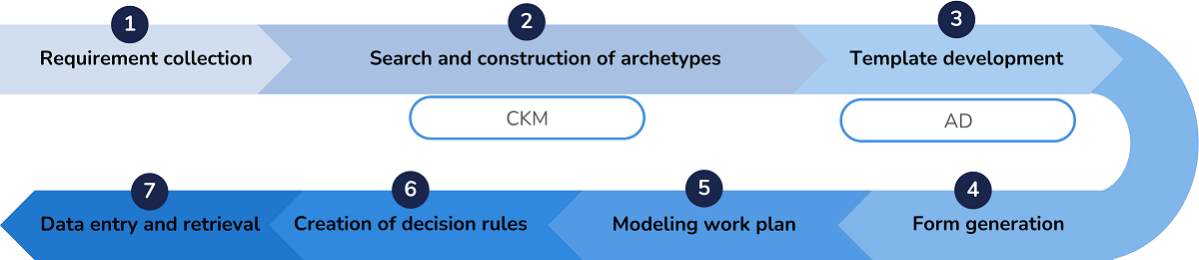
Methodological approach followed in the standardization of corneal transplantation electronic health records using openEHR specifications. AD: Archetype Designer; CKM: Clinical Knowledge Manager.

In this sense, the methodology adopted in this study uses openEHR to contribute to the ongoing efforts to improve the quality and safety of patient care through the implementation of standardized EHRs. Figure 1 represents in a simplified way the different stages of the methodology carried out in this study.

Initially, as the objective was to develop a case study on the transformation of the corneal transplantation records of a particular hospital institution, a direct line of communication with the medical team was deemed indispensable to conduct an in-depth inquiry on the necessary requirements.

After collecting the requirements, the modeling of the openEHR structures that will support the registration and management of corneal transplantation records and the timely execution of associated tasks of corneal transplantation records began, which includes archetypes, templates, forms, decision rules, and work plans.

In the next subsections, the characterization of the work developed in each of the stages that compounds the methodology represented in Figure 1 will be described in detail.

### Requirement Collection

Requirement collection is a crucial step in the development of health care systems and applications as it lays the foundation to ensure that the needs and expectations of all parties involved are understood and incorporated into the final solution.

Accordingly, the first step of the methodology started with stakeholder identification, including health care professionals and IT personnel. Overall, the process of collecting requirements for enhancing the corneal transplantation records using openEHR was a collaborative effort between medical staff, the IT personnel of the hospital’s information systems department, and the developers.

After identifying a work group, a series of meetings were organized to initiate the requirement-gathering phase. This stage entailed the active participation of stakeholders, who served as the primary source of knowledge for modeling, guiding the design and development of the openEHR structures. During these collaborative meetings, a thorough analysis of the current documentation processes and data management practices was conducted to identify areas for improvement. The meetings were structured to stimulate an active feedback process from the stakeholders regarding existing workflows, specific needs and preferences, and any identified pain points and suggestions for improvement.

Over the course of these conversations, it became apparent that the information system that the hospital used for the management of corneal transplantation records was plagued by significant shortcomings, including the possibility of errors in data entry, loss of information, difficulty sharing data between health care providers, and lack of standardization.

Therefore, the main goal was to address these issues and implement a more reliable and comprehensive information management system that used openEHR structures to mitigate the problems acknowledged. Through a collaborative effort, the stakeholders were able to delimit the scope and define the requirements and use cases of the clinical domain to be modeled.

The work group identified 5 key events in the corneal transplantation process in which data-recording actions could take place. These moments were carefully analyzed to ensure that all necessary data are captured with the highest level of accuracy and consistency. Textbox 1 provides a description of each key event.

**Description of the 5 key events identified within the corneal transplantation process.**

**Key event and description**

- Corneal transplantation proposal: to insert the data concerning the corneal transplantation proposal, such as type of transplant, laterality, diagnosis, priority, and motive, among others
- Contact for corneal transplantation: to record the 3 possible contact attempts for corneal transplantation, including information such as phone number, date of contact, result, reason, and date of next contact
- Schedule anesthesia consultation: to enter data regarding the scheduling of the anesthesia consultation; it contains the requesting service, the date of the appointment, the motive, and additional information
- Perform anesthesia consultation: to record the result of the anesthesia consultation, the executing service, the execution date of the appointment, and observations
- Manage suspended proposal: to register the result of the decision regarding suspended corneal transplantation proposals, either to reinstate to the list or to abandon, and the corresponding reason

### Modeling and Development of Archetypes, Templates, and Forms

Archetypes, templates, and forms are interconnected concepts in openEHR that work together to ensure that clinical data are consistently collected, stored in a structured and meaningful way, and retrievable in a usable format [18,21]. By providing a standard, flexible, and scalable manner to manage clinical data, they also serve as a foundation for data sharing and exchange among different health systems and organizations, thereby promoting interoperability [22].

Archetypes are reusable, modular building blocks that describe the structure and content of clinical data elements. They define the data types, units, constraints, and other properties of specific clinical concepts, such as patient demographics, test results, and medication information [19,22].

In turn, templates are collections of archetypes that define a specific clinical record, such as a patient’s progress notes, a medication prescription, or a diagnostic test result. Templates provide standardized structure and content for clinical records, ensuring that data are collected consistently in a usable and meaningful format [19].

Forms, on the other hand, provide a user-friendly interface based on templates for the input and retrieval of clinical data, supporting the entry and display of structured, semistructured, and unstructured data [23]. Forms can be customized and configured to meet the unique requirements of various clinical scenarios.

In summary, archetypes provide the building blocks for clinical data structures, templates define the standard structures for specific clinical records, and forms serve as a user-friendly interface for the input and retrieval of clinical data.

The development process of the openEHR forms in which data can be introduced in some tasks of the corneal transplantation workflow involves transforming archetypes into templates and later into forms. For representation purposes, and to simplify the demonstration of the development process, from now on the description will focus on illustrating the development of the openEHR structures, archetypes, templates, and forms related to the corneal transplantation proposal.

As previously stated, the first step involved meetings with domain experts to define the scope of the clinical domain to be modeled and collect data requirements as specified by the archetype modeling methodology [24]. The main stakeholders involved in this process were health care professionals who were familiar with openEHR, the archetype development process, and clinical terminologies.

After determining the scope of the modeling process and identifying the clinical concepts and information elements involved, the second step entailed searching the Clinical Knowledge Manager for existing archetypes that fit the scope of the modeling scenario under consideration. The Clinical Knowledge Manager is an openEHR community pillar that enables worldwide governance of domain knowledge artifacts as well as collaborative development, management, and publishing [25]. It is an open-source library of openEHR archetypes and templates that lays the foundation for both semantic and syntactic interoperability [26].

Some archetypes were used directly, whereas others did not fully represent the data elements and had to be adapted through specialization. When no corresponding archetypes existed, new ones were created. The openEHR Reference Model defines 4 major categories of archetypes: COMPOSITION, SECTION, ENTRY, and CLUSTER. A COMPOSITION is a container class, whereas a SECTION is an organizing class, both of which contain ENTRY objects [27]. The ENTRY class is further specialized into ADMIN_ENTRY, OBSERVATION, EVALUATION, INSTRUCTION, and ACTION subclasses, of which the latter 4 are kinds of CARE_ENTRY. CLUSTERS are reusable archetypes that can be used within any ENTRY or CLUSTER [16].

**Figure 2:**
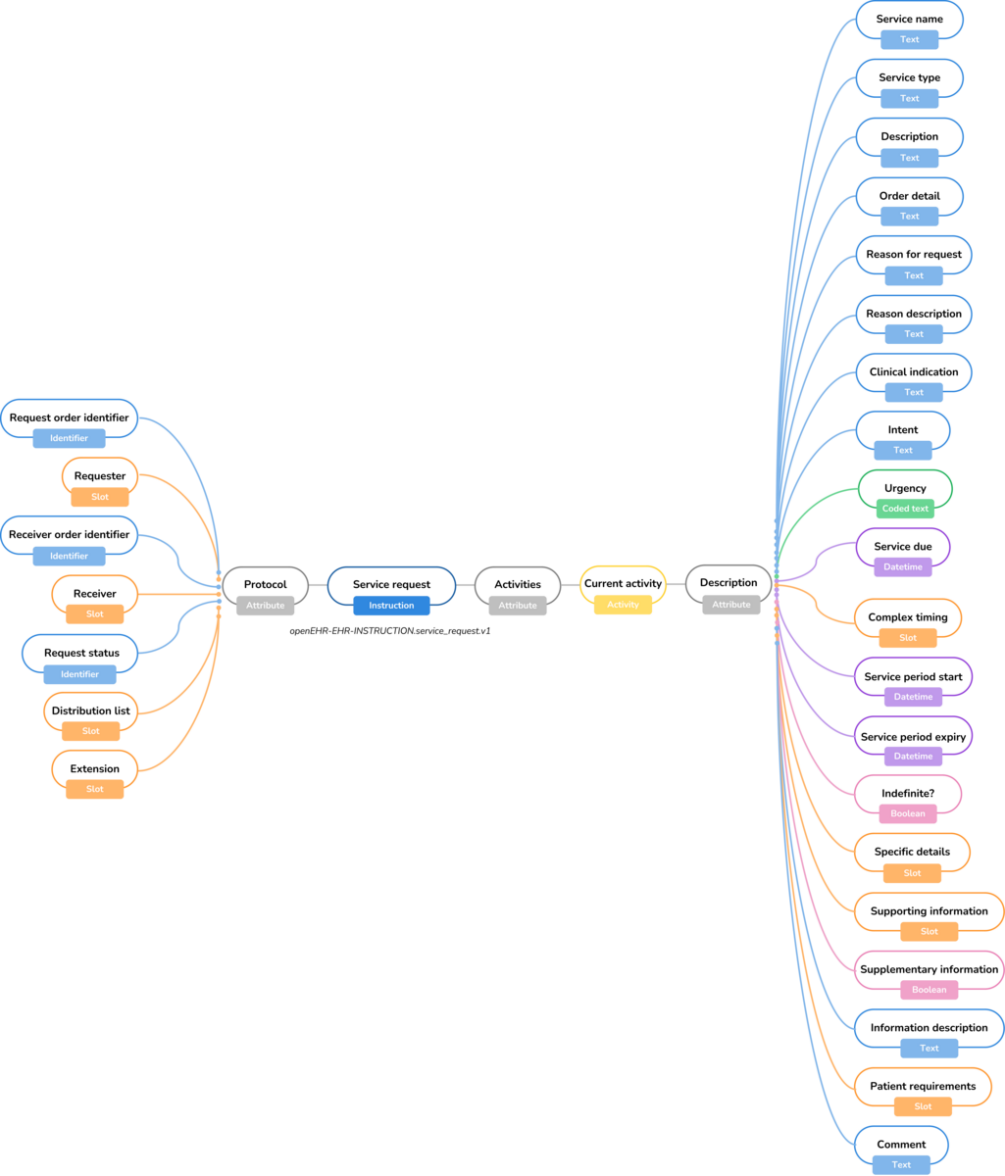
Mind map view of the “Service Request” archetype. EHR: electronic health record.

**Figure 3:**
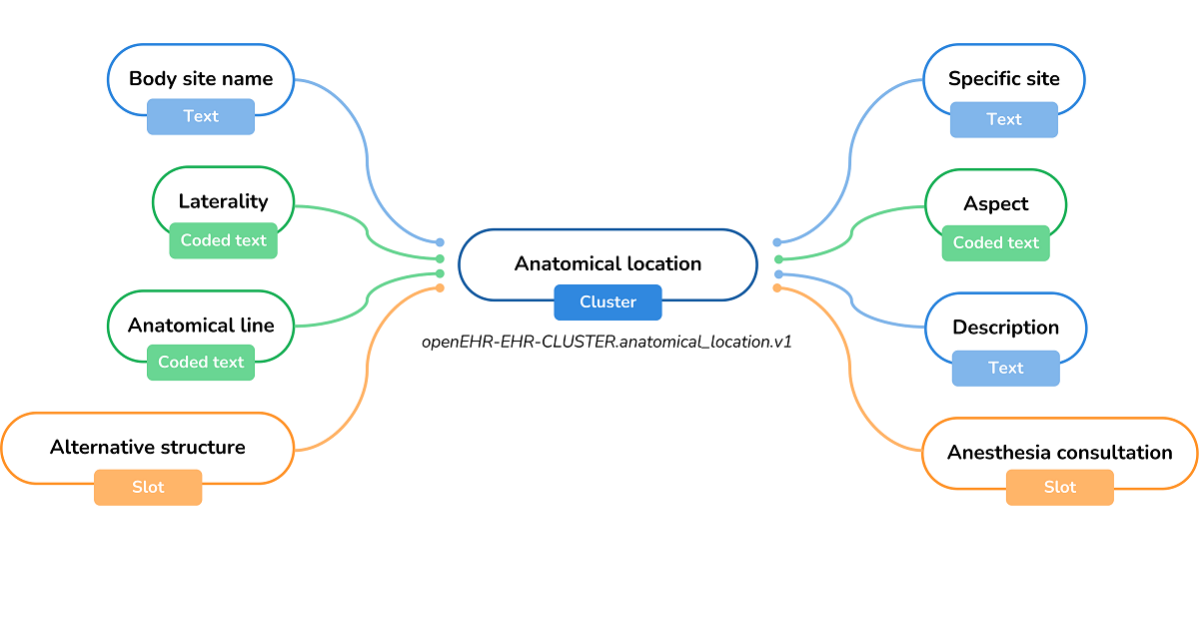
Mind map view of the “Anatomical Location” archetype. EHR: electronic health record.

For the corneal transplantation proposal, 2 archetypes were found suitable for use, namely, the service request (Figure 2), which is part of openEHR’s INSTRUCTION subclass of the ENTRY class, and the anatomical location (Figure 3), which is part of openEHR’s CLUSTER class.

**Figure 4:**
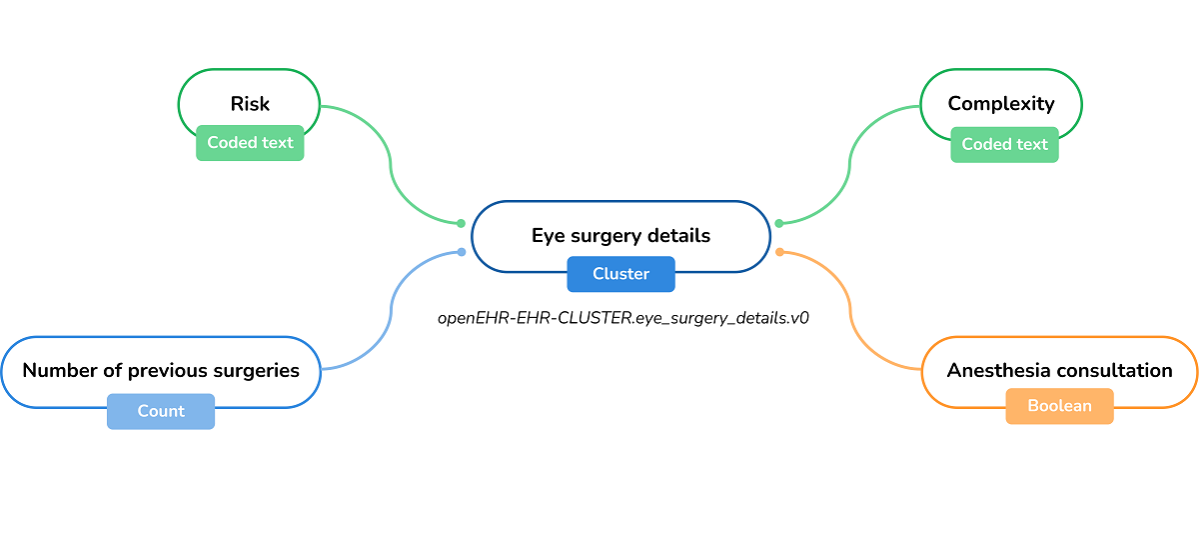
Mind map view of the “Eye Surgery Details” archetype. EHR: electronic health record.

In addition, a third archetype was created to contemplate some procedure aspects, namely, the risk, complexity, number of previous surgeries, and need for anesthesia consultation. This archetype was named “Eye Surgery Details” and belongs to the CLUSTER class. Figure 4 depicts the archetype’s mind map.

At the end of this step, information concerning applicable data constraints, such as data types, cardinality, occurrences, and specific data values (eg, terminologies for coded values and ranges for numerical values), was stipulated for each archetype.

It is worth mentioning the use of Systematized Nomenclature of Medicine–Clinical Terms terminologies for mapping the coded values of the “Laterality” item belonging to the “Anatomical Location” archetype. Local terms were used for the remaining coded text items. Table 1 provides a description of the coded values used for each coded text item.

After the discovery and development of the required archetypes, the Archetype Designer tool was used to assemble and constrain the archetypes into a template that represents the requirements of the corneal transplantation proposal. A template of the COMPOSITION type was created using the “Request for Service” archetype and named “Corneal Transplantation Proposal.” To begin, the “Service Request” archetype was incorporated into the content attribute. The template was then modified to remove items that were irrelevant to the clinical context being modeled, such as “Service Type,” “Order Detail,” and “Intent.” Both the “Anatomical Location” and “Eye Surgery Details” archetypes were imported into the “Specific Details” cluster. The items “Aspect,” “Anatomical Line,” and “Description” of the “Anatomical Location” archetype were also excluded from the template. Subsequently, some items were assigned specific default values, the content of which can be found in Table 2.

**Table 1:** Description of the coded values assigned to each coded text item.

Archetype	Item	Code	Value
Anatomical location	Laterality	SNOMED-CT^a^::362503005	Left eye
Anatomical location	Laterality	SNOMED-CT::362502000	Right eye
Anatomical location	Laterality	SNOMED-CT::362508001	Both eyes
Eye surgery details	Risk	at0004	High
Eye surgery details	Risk	at0005	Moderate
Eye surgery details	Risk	at0006	Low
Eye surgery details	Complexity	at0007	High
Eye surgery details	Complexity	at0008	Moderate
Eye surgery details	Complexity	at0009	Low
Service request	Diagnosis	H16319	Corneal abscess
Service request	Diagnosis	H1830	Corneal membrane alterations
Service request	Diagnosis	H52219	Irregular astigmatism
Service request	Diagnosis	H18739	Descemetocele
Service request	Diagnosis	H18519	Corneal endothelial dystrophy
Service request	Diagnosis	H18719	Corneal ectasia
Service request	Diagnosis	H1820	Corneal edema
Service request	Diagnosis	T868499	Corneal graft (complication)
Service request	Type of transplant	08R83KZ_tt_d	Total transplantation (right eye)
Service request	Type of transplant	08R83KZ_tt_e	Total transplantation (left eye)
Service request	Type of transplant	08R83KZ_dalk_d	Anterior transplantation—DALK^b^ (right eye)
Service request	Type of transplant	08R83KZ_dalk_e	Anterior transplantation—DALK (left eye)
Service request	Type of transplant	08R83KZ_dsaek_d	Anterior transplantation—DSAEK^c^ (right eye)
Service request	Type of transplant	08R83KZ_dsaek_e	Anterior transplantation—DSAEK (left eye)
Service request	Type of transplant	08R83KZ_dmek_d	Anterior transplantation—DMEK^d^ (right eye)
Service request	Type of transplant	08R83KZ_dmek_e	Anterior transplantation—DMEK (left eye)
Service request	Urgency	at0136	Emergency
Service request	Urgency	at0137	Urgent
Service request	Urgency	at0138	Routine

^a^SNOMED-CT: Systematized Nomenclature of Medicine–Clinical Terms.

^b^DALK: deep anterior lamellar keratoplasty.

^c^DSAEK: Descemet stripping endothelial keratoplasty.

^d^DMEK: Descemet membrane endothelial keratoplasty.

**Table 2:** Description of the default values assigned.

Archetype	Item	Default value
Service request	Service name	Corneal transplantation
Anatomical location	Body site name	Eye
Anatomical location	Specific site	Cornea

**Figure 5:**
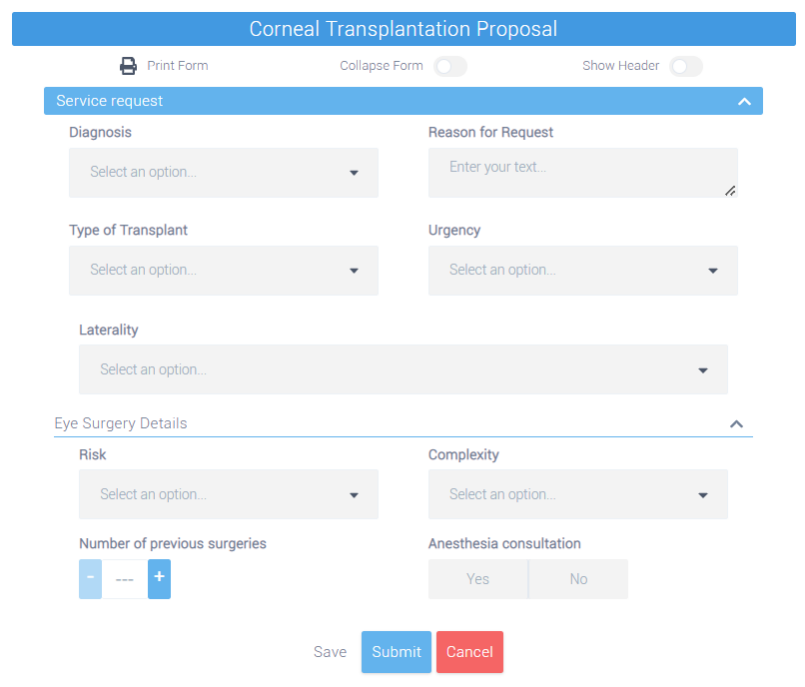
Graphic representation of the user interface form generated from the “Corneal Transplantation Proposal” template.

To facilitate interpretation and manipulation, the template was exported in the Operational Template structure and later converted into the JSON Data Template structure. Finally, the JSON Data Template was injected into the Form Builder tool to format the user interface form, which can be consulted in Figure 5.

At the end of this stage, 5 forms were created. To simplify the identification of each form, Textbox 2 assigns an identification label to each form. Following that, each of these forms will be associated with specific tasks in the corneal transplantation workflow, acting as storage schemes for different patient tasks. A more detailed description of the deployment will be provided later in this paper.

**Forms developed for the corneal transplantation workflow.**

**ID and form**

- F1: corneal transplantation proposal
- F2: contact for corneal transplantation
- F3: schedule anesthesia consultation
- F4: perform anesthesia consultation
- F5: manage suspended proposal

### Work Plan Modeling

In recent years, a major extension has been incorporated into the openEHR specifications for addressing requirements in the area of clinical process automation, known as Task Planning [18]. Task Planning allows for the management of standardized Task Plans (TPs) and clinical workflows. The main concept of Task Planning is centered on a plan, or set of plans, that is devised to accomplish a specific goal and pertains to an active subject [28].

Within the Task Planning specification, in terms of conceptual elements, the formal concept at the highest level of hierarchy is the Work Plan (WP), which encompasses one or more TPs [29]. A WP defines a series of tasks that need to be performed in a specific order to achieve a clinical goal with respect to a subject, human, or other subject of care [18]. It is worth noting that, as the WP is subject-centric, each subject requires a unique instance of a WP. WPs can organize and monitor the progress of clinical tasks, ensuring that all necessary steps are taken in a timely and efficient manner [29]. In turn, each TP incorporated within a WP is an explicit depiction of the work that must be performed in a particular work context by the principal performer, along with other possible participants [18]. In openEHR, the principal performer refers to the individual or entity responsible for carrying out a specific clinical action, which enables the tracking of clinical actions back to their responsible parties. The data collected through forms and the decision support provided by Decision Logic Modules (DLMs) can be used to define and update WPs in real time.

In this study, a WP regarding corneal transplantation was modeled to ensure that the implementation of the solution proceeds smoothly and is completed within the specified timeline.

The first TP defined in this WP concerns the corneal transplantation proposal. This task is available to certain ophthalmologists in specific contexts. In total, 2 TPs related to the anesthesia consultation were also included: scheduling and carrying out the consultation, which are assigned to different groups of professionals. Finally, 2 more TPs were modeled: one related to the contact for transplantation carried out by administrative staff and one related to the performance of the transplant, which is allocated to the physician who submitted the proposal in the first instance.

**Figure 6:**
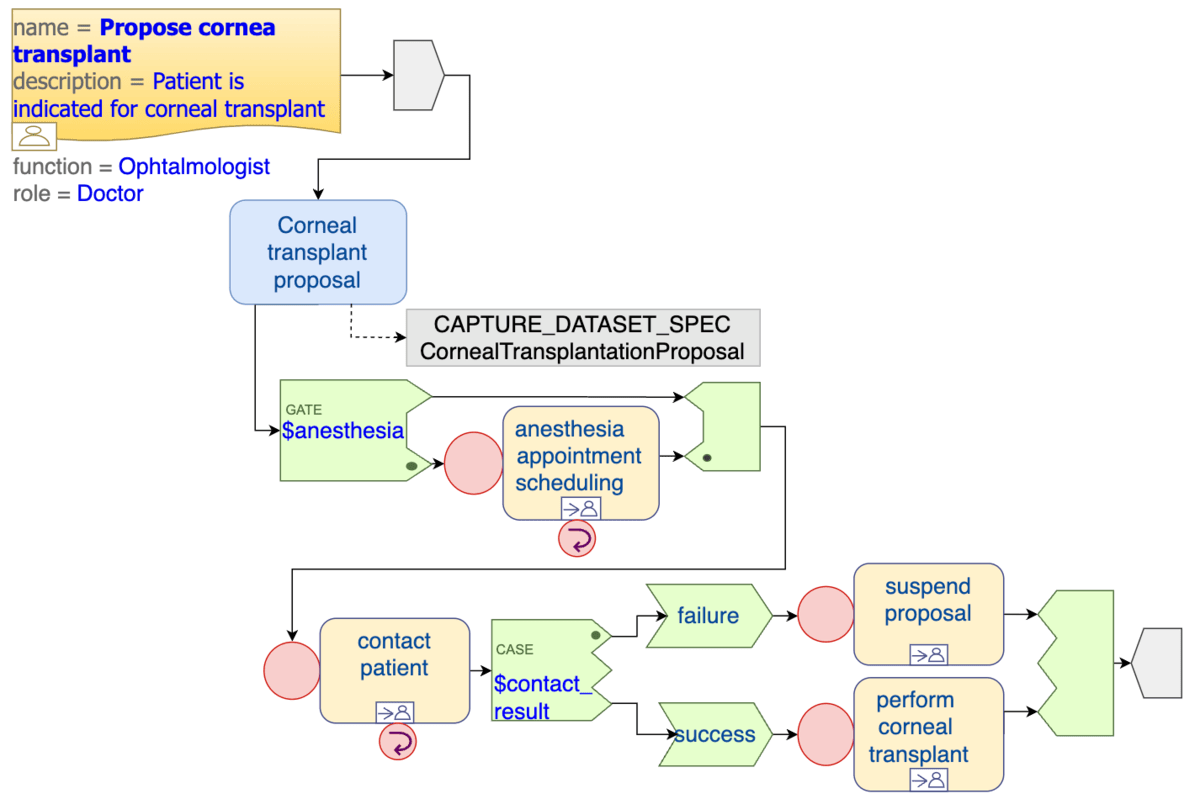
Representation of the top-level Task Plan.

Figure 6 shows the first TP in a simplified way. It is interesting to note that, in this first TP, the proposal form for a corneal transplant is completed in the first performable task. After submission, the execution of the anesthesia consultation relies on the value assigned to the DV_BOOLEAN field labeled “Anesthesia Consultation” within the “Corneal Transplantation Proposal” form. If this field indicates a true value, the subsequent steps for that patient involve scheduling the anesthesia consultation; conducting the consultation; and, eventually, establishing contact for the transplantation procedure. On the contrary, if the anesthesia consultation is not necessary, the patient goes to the active waiting list represented by the cornea transplant contact tasks. If the contact is successful, the patient proceeds to undergo the transplant; if not, the proposal is suspended, and the suspension management task becomes available. All dispatchable tasks presented in this example are connected to other TPs, which mainly have a performable task with one of the associated modeled forms.

Later in this paper, the main decisions that need to be made in the course of the WP will be explained.

### Construction of Decision Rules

#### Overview

Clinical decision support is a key component of the openEHR architecture, responsible for automating and enhancing decision-making. The openEHR community defines decision rules and guidelines using a specific syntax, the Decision Language (DL) [30]. DL is a formal language for representing clinical knowledge in decision-making and expressing decision support logic through rulesets.

DLMs are multisectioned modules with a specific structure for defining rules, encompassed in DL. DLMs are computerized decision-making instructions that provide a standardized and automated way to apply decision rules to patient data within the EHR in real time [30,31].

DLMs enable health care organizations to implement algorithms and rules to determine the best course of action for a given patient [32]. The output from a DLM can be used to guide clinical decision-making; provide alerts or notifications to health care providers; or drive automated actions within the EHR, such as ordering tests or medications [18].

To ensure the efficiency of the solution, decision rules were established for forms and TPs to define the logic and actions that should be taken based on specific data inputs. The rules are stored in a standardized structure that can be applied to patient data at runtime.

Overall, by automating the application of decision rules, DLMs can help reduce the risk of errors and variability in decision-making, providing more consistent care [33].

#### Form Decision Rules

The DLMs allow the forms to automatically adapt to the specific constraints for a given patient or scenario and to the responses of the health care professional filling in the form fields, ensuring that the information entered is always consistent with established clinical guidelines and best practices. The data collected through the forms are used as input for DLMs to support the delivery of real-time decision support.

By automating the process of adjusting forms to specific scenarios and inputs, the solution can provide decision support to health care providers, guiding them through the process of entering data and making clinical decisions.

Hence, according to the requirements gathered in the first stage of the methodology, 2 DLMs were created for guiding health care professionals in the process of filling in the forms F1—“Corneal Transplantation Proposal” and F2—“Contact for Corneal Transplantation.” Table 3 describes the association among the forms, DLMs, and rules.

**Table 3:** Association among forms, Decision Logic Modules (DLMs), and rules.

Form	DLM	Rule	Rule description
F1	1	1	If the patient’s age is ≥75 years, anesthesia is mandatory.
F2	2	1	If the patient intends to leave the transplant list, the motive is mandatory.
F2	2	2	If the patient needs to postpone the contact, the next contact is mandatory.

The rules associated with each DLM, including the conditions necessary to trigger a certain action, are described below.

For the “Contact for Corneal Transplantation” form, the decision logic operates as follows: If the “Result” field is set to “Cancellation,” the “Motive” field becomes mandatory. Similarly, if the “Result” field is set to “Postpone contact,” the “Next contact” field becomes mandatory.

Regarding the “Perform Anesthesia Consultation” form, the logic dictates that if the “Age” field has a value of 75 or greater, the “Anesthesia” field must be set to “Yes.”

#### WP Decision Rules

The DLM rules built to support the necessary decisions in the modeled WP were crucial for the correct functioning of the respective materializations.

The first rule to be processed concerns the decision whether the patient should proceed to an anesthesia appointment. If the priority of the proposal is urgent, the patient does not need an anesthesia appointment. If it is not urgent, the need for an anesthesia consultation is decided by the “Anesthesia” field filled in by the physician in the “Corneal Transplantation Proposal” form.

Regarding the anesthesia consultation, a rule was created to verify the success of the consultation and, thus, decide whether the patient goes to the contact for transplantation. This rule uses the data entered by the anesthesiologist in the respective form.

**Figure 7:**
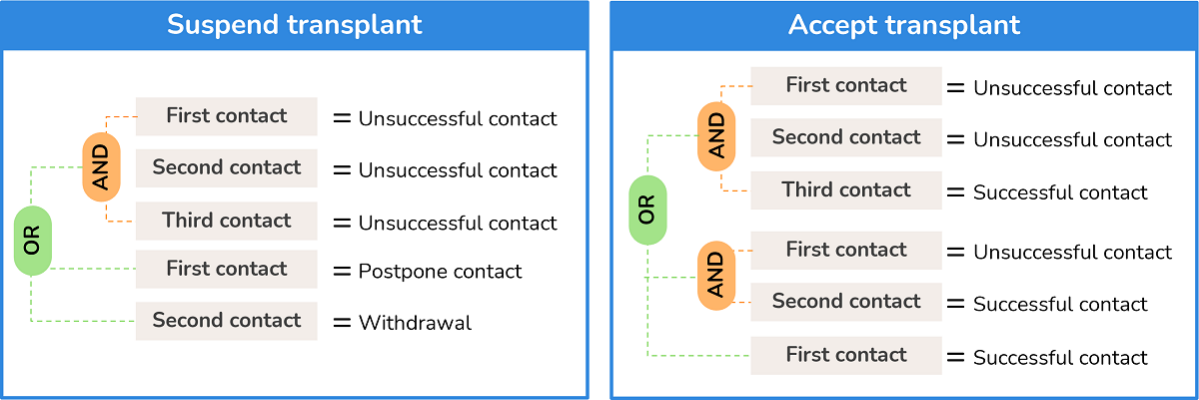
Decision logic associated with the Work Plan.

Then, the most complex rule was built to support the decision after the patient is called for transplant. Figure 7 represents the logic behind the decision to be made after the transplant contact, where up to 3 contacts with the patient can be registered.

Finally, there is still a decision that is made for suspended patients, which is based on whether they are removed from the transplant waiting list and the WP ends or the patient is reinserted into the transplant contact list, making the respective performable task available.

### Ethical Considerations

In this study, all patient demographic information was anonymized through the use of the openEHR separation of the Demographic Information Model and Clinical Information Model. This approach ensures that personal identifiers are not linked to clinical data, thereby maintaining patient confidentiality and minimizing the risk of reidentification. Furthermore, the data analysis conducted in this study focused on performing statistical analyses at an administrative level. This includes examining the quantity of tasks available, the completion rate of these tasks, and referencing solely the number of patients enrolled in the study. The analysis was restricted to aggregate data, ensuring that individual patient identifiers and clinical details were not disclosed. This methodological approach allowed for a comprehensive evaluation of the operational aspects of the study without compromising patient confidentiality or violating ethical standards. The data structure of the generated forms can be found [34]. Given these protections, this study qualified for an exemption from ethical review, as per the Code of Ethical Conduct of the University of Minho.

## Results

### Deployment and Architecture Overview

The openEHR ecosystem provides a comprehensive solution for managing health care data by combining different tools and technologies. In openEHR, forms, DLMs, and WPs interact in a complementary manner to support the provision of effective and efficient clinical care. These modules ensure that patient data are accurate, consistent, and accessible and provide data validation, automated actions, and real-time decision support to ensure that the best course of action is taken for each individual patient. Furthermore, the integration of these modules allows for the organization and tracking of the progress of clinical tasks to ensure that all necessary clinical tasks are performed in a timely and efficient manner.

Once the modeling and validation of all openEHR structures supporting corneal transplantation EHRs were concluded, it was necessary to integrate them into an automated solution. The implementation step involved the integration of a set of tools from the openEHR ecosystem, which include Form Builder, the TP engine, and the DLM engine.

Form Builder is a web application that is designed to generate user interface forms from openEHR templates. It provides a platform for health care professionals who possess modeling knowledge to customize the user interface forms by adjusting the formatting, such as choosing colors and fonts and determining whether fields should be shown or hidden. In addition, it allows for the association of functions and refsets to form fields, which can add further functionality to the form. Using Form Builder, health care professionals can streamline the process of data entry by creating forms that are intuitive and optimized for their specific clinical workflows.

In turn, the TP engine is a tool that manages all workflows modeled by the professionals, including materialization, task status, decision management, and allocation of performers. To accomplish this, the TP module defines a formal model for processing tasks and workflows. This tool is designed to translate graphical workflow models into executable models of an organized plan that, when carried out by an engine, notifies employees of tasks. Overall, the TP engine provides an automated solution for managing and executing complex workflows in the health care setting.

The DLM engine, on the other hand, is a decision logic engine that is responsible for processing clinical or operational rules and triggering specified events based on predefined conditions. This engine plays a crucial role in supporting the logic of forms and TPs. Accordingly, it receives requests from both Form Builder and the TP engine. It ensures that decision rules are executed correctly and consistently, leading to improved patient care and outcomes.

**Figure 8:**
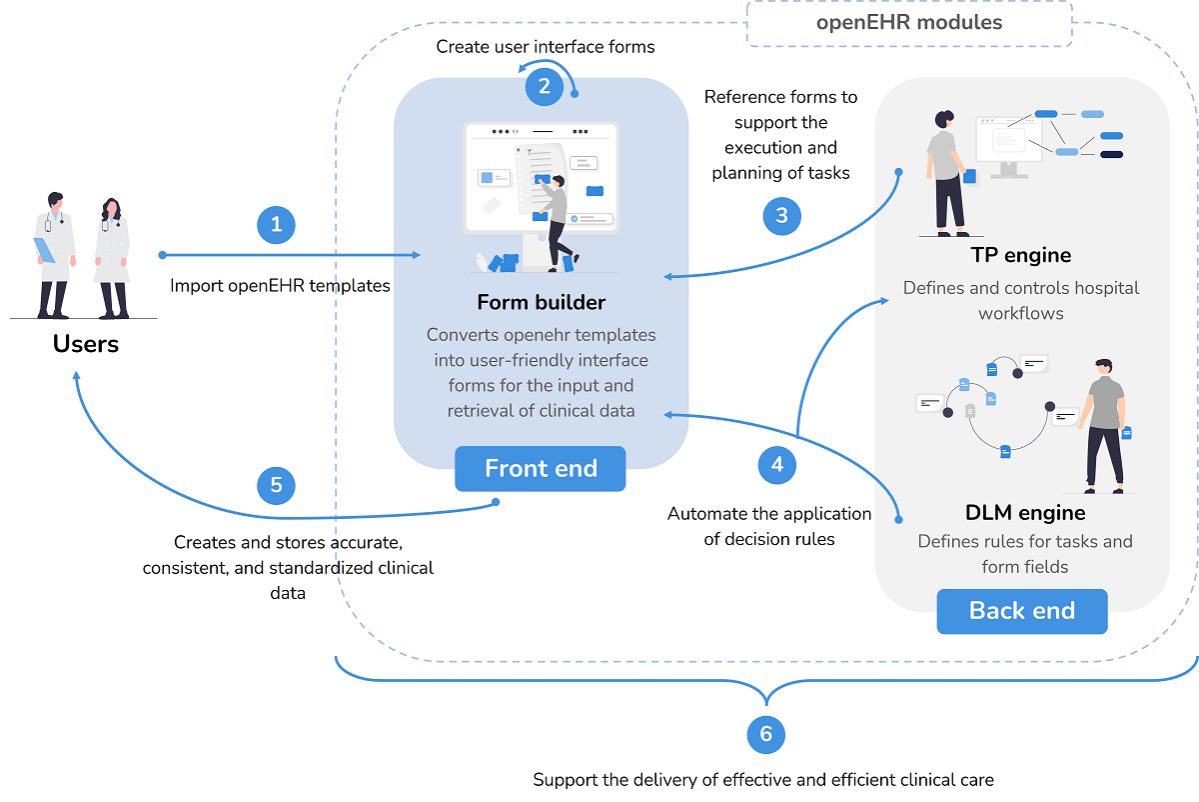
Representation of openEHR modules and their interactions. DLM: Decision Logic Module; TP: Task Plan.

Figure 8 illustrates the different interactions that occur among these openEHR modules.

The integration of all these components was also explored in this research.

**Figure 9:**
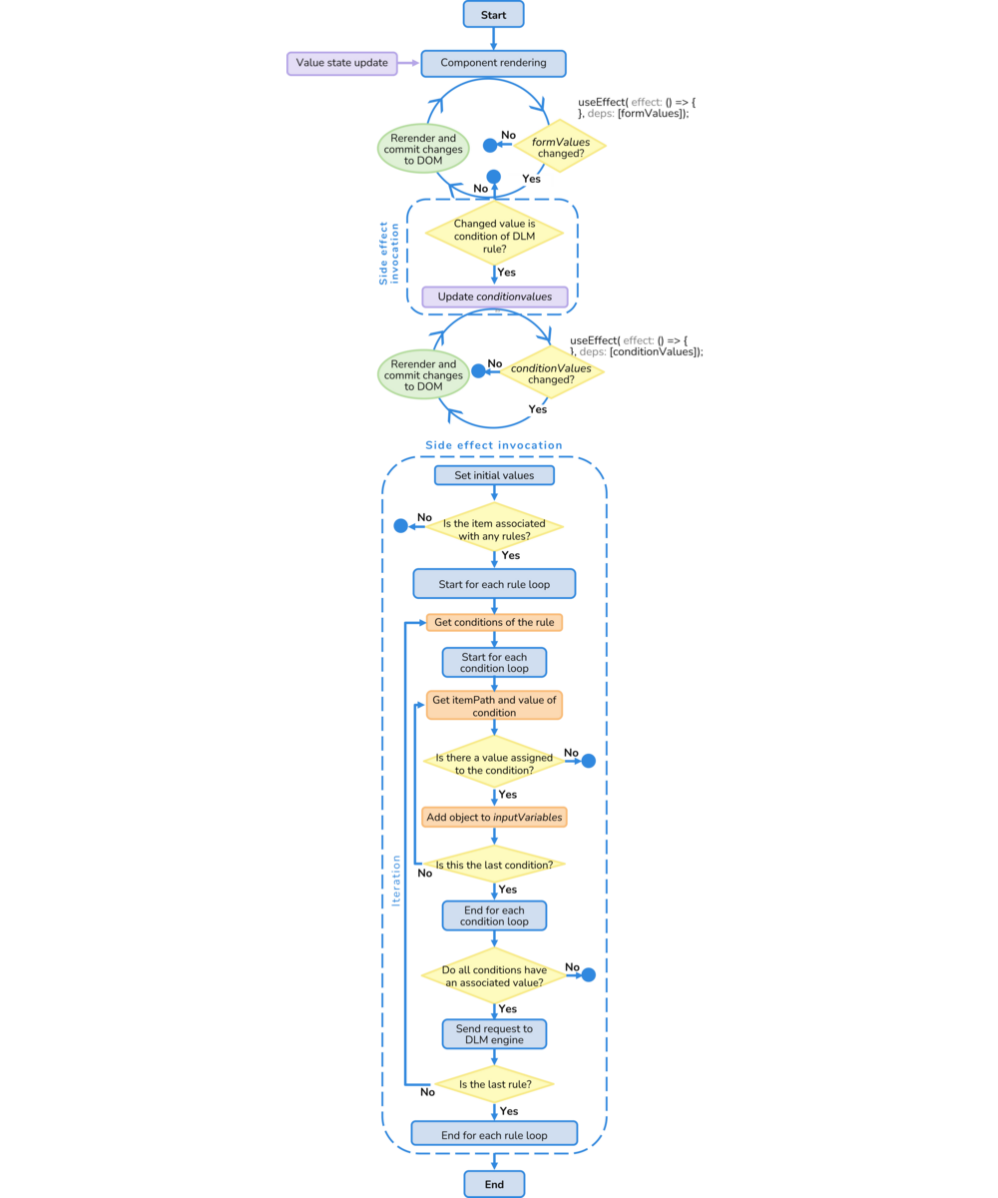
A schematic illustration of the logic flow that occurs in the Form Builder process responsible for initiating rule requests. DLM: Decision Logic Module; DOM: Document Object Model.

Regarding the integration of Form Builder and the DLM engine, the process starts when a health care professional updates a form field. Whenever the value of a field changes, Form Builder performs an internal processing to check whether that field is a condition of any rule within the DLM associated with the current form. If so, Form Builder identifies each associated rule and, for each of them, verifies whether there are more associated conditions that already have a value assigned to them. When all the conditions of a rule have an assigned value, Form Builder triggers a POST request to the DLM engine with the input variables. This is a well-defined sequence of computational tasks that need to be followed to properly implement the specific set of rules that have been modeled for each form. For better understanding, a graphical representation of the processing that occurs in Form Builder to verify the need to trigger any rule requests is presented in Figure 9.

**Figure 10:**
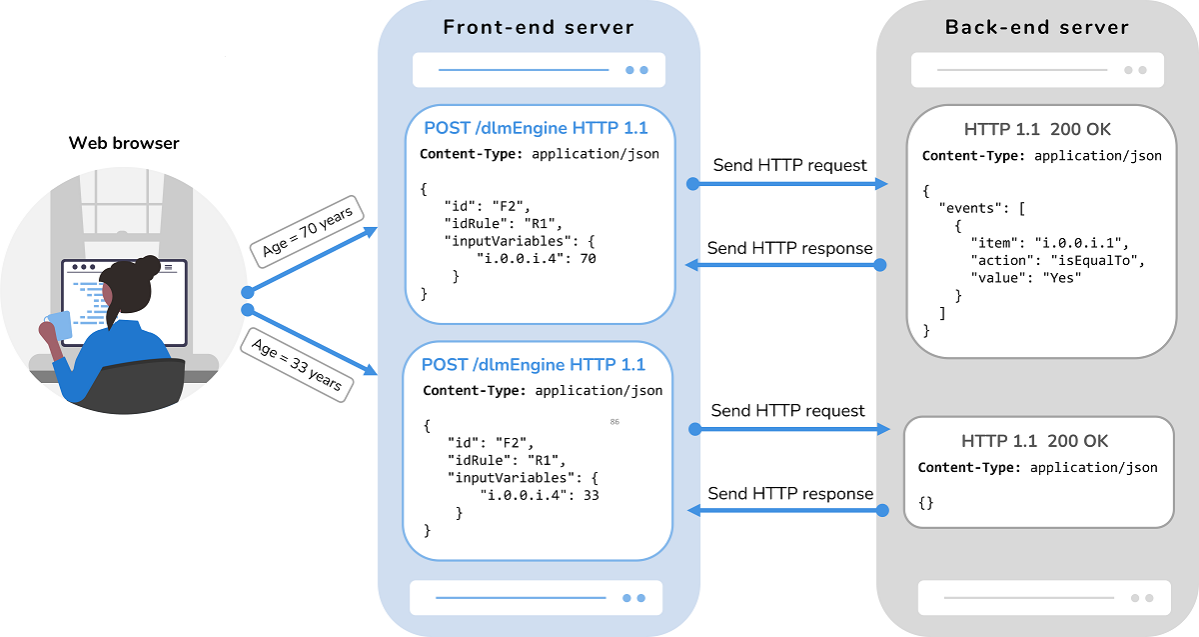
Diagram of HTTP request and response interactions between Form Builder, which acts as the front-end server, and the Decision Logic Module (DLM) engine, which serves as the back-end server, in 2 distinct settings.

Upon receiving the input variables, the DLM engine checks whether the conditions are met for executing a given rule. If the values assigned to the form fields do not fulfill a rule, an empty object is returned. On the other hand, if the values meet the conditions for executing a rule, the engine returns the path of the affected field and the type of event to execute. Form Builder then triggers the necessary actions for each field depending on the response. Figure 10 serves as an exemplification of the HTTP requests that are exchanged between Form Builder (front-end server) and the DLM engine (back-end server) in 2 distinct scenarios. In the first scenario, the health professional enters a value that triggers the execution of a rule, whereas the second scenario does not involve the activation of any rule. This diagram provides a clear representation of the data flow and communication between the 2 servers during the execution of the rule-based system.

The integration of the DLM engine with the TP engine represents a remarkable achievement in the openEHR ecosystem. The successful interaction between the 2 enables the handling of all conditional structures encompassed in a WP, including condition groups, decision groups, and event groups, with the support of the DLM engine. In general, when the path of a WP materialization reaches a decision point, a request is issued to an application programming interface provided by the DLM engine with the variable associated with that point. In turn, the DLM engine processes the rules and conditions associated with the request that was made and returns a response. Through the response received, the TP engine manages to associate it with the respective branch (decision branch, condition branch, or event branch) and proceed with its execution.

In the openEHR ecosystem, the TP engine can refer openEHR forms to support the planning and execution of hospital tasks. By providing a user-friendly interface for entering and retrieving data, Form Builder makes forms available to health care professionals that can be used to collect data required for various tasks, and it ensures that the collected data are accurate and standardized. Furthermore, the responses given by health care professionals in certain fields of the form can be determinant to define which path should be triggered after submitting the form. By integrating the TP engine and Form Builder, health care providers can ensure that relevant information is captured and acted upon as part of the patient’s care plan, leading to improved patient outcomes and increased efficiency in health care delivery. This integration enhances the quality of care provided to patients and enables health care professionals to make informed decisions based on accurate and timely data.

After successfully integrating these components in a symbiotic IT environment, it was possible to install the solutions in the Portuguese hospital to implement and evaluate them in a real-world setting.

For several years, this hospital has maintained a collaborative relationship with the research center where the working group is based. Given this existing partnership, the hospital was deemed an ideal site to deploy and assess the newly developed solution.

Considering the current IT infrastructure of the hospital, the amalgamation of the proposed solution was not hampered by any intricate integration challenges and unfolded as a straightforward process. To streamline the workflow and improve efficiency, Form Builder was integrated into a web application already implemented in the hospital that presents a detailed listing of the tasks assigned to a certain user. This integration enables health care professionals to view a comprehensive list of assigned tasks and submit completed forms directly through the portal. The TP engine is the mechanism that controls and triggers the tasks made available to the medical team on the professional portal. As a result, the professional portal serves as a centralized hub for task management and data collection, enhancing the overall clinical workflow.

To ensure that only authorized health care professionals had access to corneal transplantation tasks, it was necessary to create specific members for this purpose within the demographic of professionals with their respective capabilities, roles, and functions. Once the members of each team were established, they were associated with the corresponding tasks. By establishing a clear hierarchy of roles and responsibilities, the hospital was able to ensure that the tasks related to corneal transplantation were being accessed and completed by qualified and authorized personnel.

The collaboration between the health care institution and the research center has paved the way for the exchange of knowledge and resources, allowing for a more efficient and effective implementation of the solution. Furthermore, this cooperation has fostered a culture of innovation and continuous improvement in the hospital’s clinical practices, ultimately yielding beneficial outcomes for the patients.

The main challenges encountered during deployment were related to the lack of health care professionals with knowledge of openEHR and modeling skills. As a result, the team conducted several demonstrations and provided comprehensive documentation to facilitate the users’ adoption of the tools. Despite these challenges, the feedback and acceptance from medical staff were generally positive as they reported ease of adaptation and expressed satisfaction with the provided tools.

### Statistical Analysis

In a data-driven world, statistical analysis has become critical to gain insights and draw conclusions that may not be immediately apparent through simple visual inspections. It holds particular significance in the realm of health research, where it can be used to evaluate the effectiveness and efficiency of IT solutions such as EHRs, telemedicine platforms, and other digital health tools. In this way, health care professionals can identify areas for improvement, including the streamlining of workflows, the enhancement of usability, and the resolution of technical glitches, ultimately leading to improved patient outcomes and enhanced quality of care.

Overall, the importance of data analytics in the health care domain cannot be overstated as it has the potential to significantly impact the lives and well-being of countless individuals. Hence, to verify the efficiency and performance of the solutions offered to manage the corneal transplantation process, this section presents an analysis of the data gathered over the period of study from May 1, 2022, to March 31, 2023. This analysis will include relevant indicators and charts. Before presenting the data analysis, a brief overview of the data collection process will be provided.

The data collection process for this study required careful planning, attention to detail, and adherence to ethical guidelines to ensure the accuracy and validity of the data. First, the databases and tables of interest were selected. Then, an anonymization process was carried out to preserve the identity and privacy of the patients, which involved removing any personal identifying information from the data. Once the data had been anonymized, the relevant SQL queries were developed to extract the data required. Finally, after the data had been extracted, they were meticulously organized into descriptive statistics in the form of indicators, charts, and graphs to ease interpretation and help convey the findings.

**Figure 11:**
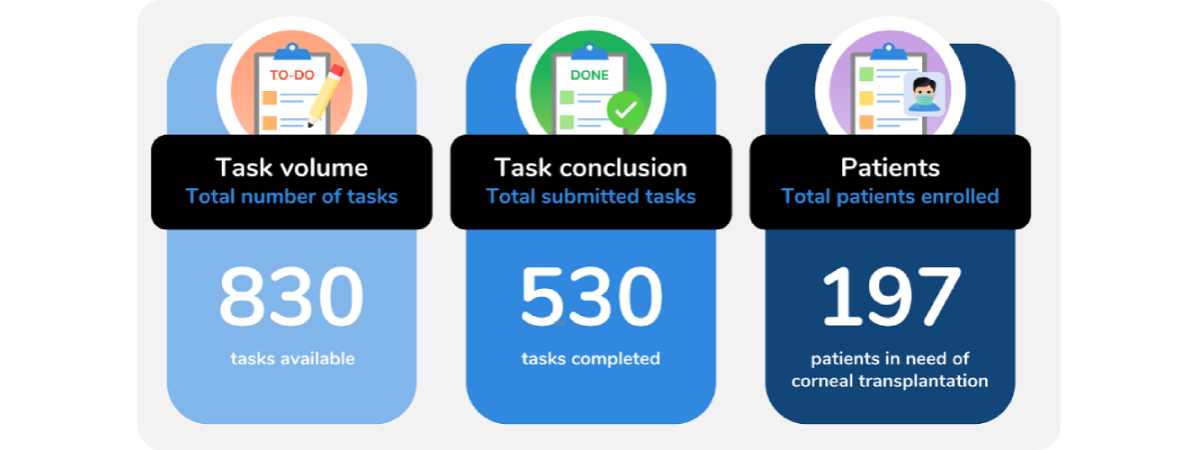
Single-number indicators of the corneal transplantation process, including task volume, task conclusion, and patient enrollment.

Figure 11 shows the graphical representation of key indicators in the corneal transplantation process, including task volume, task conclusion, and patients enrolled. The task volume represents the number of tasks available for health care professionals to fill out the corresponding forms, whereas the task conclusion corresponds to the number of tasks successfully submitted by health care professionals. On the other hand, patient enrollment indicates the number of patients registered in the corneal transplantation list.

The proportion of concluded tasks in comparison to the number of available tasks provides a clear and concise overview of the overall task completion rate and offers a general picture of how well tasks are being managed and completed. The number of available and completed tasks can help gauge the workload of health care professionals and assess the capacity of the system to handle the demands of the workflow. A higher number of completed tasks relative to available tasks indicates that the system is functioning efficiently and effectively. Meanwhile, a lower completion rate could suggest potential bottlenecks or areas for improvement in the system’s design or implementation. In this study, there is 63.9% (530/830) of concluded tasks and 36.1% (300/830) of available tasks.

The total number of corneal transplantation forms submitted by health care professionals over time can be consulted in Table 4, allowing for the identification of trends and patterns in form submission and providing insights into the volume and frequency of tasks completed.

**Table 4:** Total number of corneal transplantation tasks submitted over time (n=530).

Month and year	Corneal transplantation tasks submitted, n (%)
March 2023	33 (6.2)
February 2023	19 (3.6)
January 2023	79 (14.9)
December 2022	53 (10)
November 2022	32 (6)
October 2022	87 (16.4)
September 2022	39 (7.4)
August 2022	49 (9.3)
July 2022	30 (5.7)
June 2022	58 (10.9)
May 2022	51 (9.6)

As the corneal transplantation workflow is a complex process involving the completion of a variety of tasks, Table 5 helps visualize the distribution of these tasks across the different form categories. This table displays both the numerical and percentage distribution of submitted tasks according to each form category.

**Table 5:** Total number of corneal transplantation tasks submitted over time (n=530).

Tasks	Corneal transplantation tasks submitted, n (%)
Corneal transplantation request	216 (40.8)
Schedule anesthesia consultation	114 (21.5)
Perform anesthesia consultation	82 (15.5)
Corneal transplantation call	115 (21.7)
Manage suspended requests	3 (0.6)

**Figure 12:**
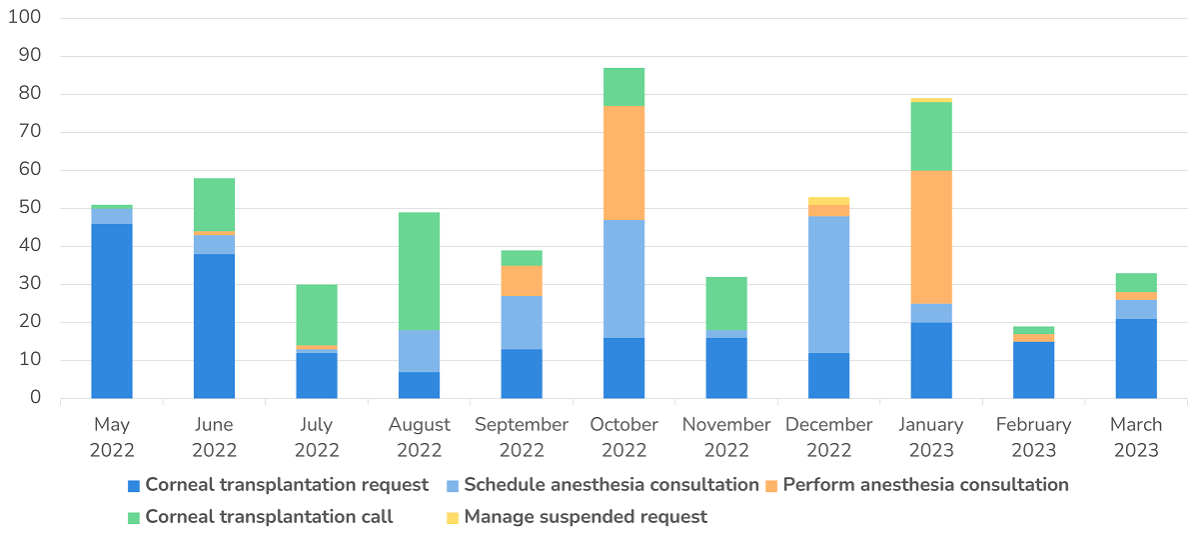
Number of submitted tasks within the corneal transplantation workflow for each form over time.

Finally, to provide a comprehensive visualization of the number of tasks submitted over time for each form category, a stacked bar chart was used in Figure 12. This chart displays the total number of tasks completed and submitted for each form category over the period under consideration, helping assess the relative contributions of each form category to the overall workflow.

## Discussion

### Principal Findings

The findings of this study offer a comprehensive and in-depth overview of the incorporation of various components of openEHR specifications, particularly the interaction of openEHR forms, WPs, and DLMs, which have not yet been fully covered in scientific literature.

To evaluate the impact of the solution, the openEHR structures were incorporated into a workflow and integrated into a health care institution. This study demonstrated the effects of the intervention on the corneal transplantation workflow, which was previously characterized by inadequate automation and an intensified risk of data loss. The use of the new technologies has the potential to substantially influence the workload of health care professionals and, consequently, affect patient care outcomes. Therefore, in addition to providing a thorough description of the implementation process, this paper also presented a statistical analysis of its effects.

The indicators presented in the *Statistical Analysis* section, which are shown in Figure 11, show the number of available tasks, completed tasks, and patients enrolled in the corneal transplantation list during the time frame of this study.

The number of available tasks was 830, which represents the total number of tasks that need to be completed. Of these tasks, 530 were completed, indicating that the completion rate of the tasks was 63.9% (530/830).

The number of available and completed tasks can help gauge the workload of health care professionals and assess the capacity of the system to handle the demands of the workflow. A higher ratio of completed tasks to available tasks indicates that the system is working efficiently and effectively. A lower completion rate, on the other hand, may suggest potential bottlenecks or areas for improvement in the system’s design or implementation.

As a result, a 63.9% (530/830) completion rate falls short of expectations. This lower rate could be explained by a number of factors. First, the hospital may have limited resources to complete the tasks within the workflow, leading to delays in completing the tasks. Furthermore, the health care professionals responsible for completing the tasks within the workflow may not have received enough training or may be inexperienced, which may imply some reluctance in adopting the new technologies.

Because most of the available tasks within the studied workflow regarded the “Contact for Corneal Transplantation,” which represents the active waiting list for corneal transplantation, the workflow completion depended on external factors such as patient health and availability of donor organs. These dependencies directly affected the time required to complete the tasks and, consequently, the total workflow.

Due to the complexity of the corneal transplantation process, a multidisciplinary service team is required, namely, the anesthesia team and the ophthalmology team, which can cause communication issues between the teams and, as a result, delays in task completion.

These factors may also explain the fluctuations in the number of task submissions over time depicted in Table 4. Hence, as a result of the influence of these factors, it was not possible to establish patterns in the data over time as expected given the nonseasonal nature of the data.

Furthermore, the enrollment of 197 patients is a significant aspect of the data as it serves as a critical contextual element in assessing the workload. However, it should be noted that the number of enrolled patients, as previously mentioned, does not accurately reflect the total number of corneal transplantation proposals submitted. This is because a single patient may be registered multiple times to undergo different procedures. As a result, the number of enrolled patients may not be a direct indicator of the workload or the number of tasks that need to be completed in a corneal transplantation workflow.

In this sense, to gain a more comprehensive understanding of workload management within a hospital setting, a more specific study on the different types of tasks available was required. This analysis enables more detailed scrutiny of the specific challenges and constraints associated with each task type and allows for the identification of more targeted solutions to enhance efficiency and productivity.

A quick look into Table 5 reveals a higher rate, 40.8% (216/530), of completed tasks associated with the “Corneal Transplantation Proposal” form in comparison to the remaining tasks. This rate can be explained by the lack of resources to perform the transplants at a quicker pace. It is also worth noting that the form with the lowest submission rate was “Management of Suspended Proposal,” indicating the fewer cases in which the corneal transplantation proposals were suspended.

Finally, Figure 12 depicts the number of submitted tasks according to each form over time. In the first months, it is possible to observe that the tasks associated with the “Contact for Corneal Transplantation” form increased, whereas the number of “Corneal Transplantation Proposal” tasks decreased. Since August 2022, a higher number of submitted tasks pertaining to the “Schedule Anesthesia Consultation” form can be observed. As a result, a month later, it is possible to observe an increase in the number of tasks pertaining to the “Perform Anesthesia Consultation” form.

The findings of this analysis emphasize the importance of effective workload management within a hospital setting. By monitoring and analyzing the number of tasks available and completed, hospitals can identify areas for improvement and ensure that patient care is not jeopardized. In addition, this study can help identify potential bottlenecks and areas of inefficiency in current workflows, informing the design and implementation of targeted interventions to enhance the effectiveness of health care operations.

### Limitations

A paper outlining the implementation steps of clinical standards in a hospital setting is a valuable resource for identifying best practices and areas for improvement, as well as for advancing patient care. However, it is important to acknowledge its potential limitations to fully understand its impact on health care.

Resource limitations are an important concern to consider as the implementation of openEHR specifications requires staff time and training, which can pose additional challenges for health care organizations. One difficulty encountered in this study was the staff workload as the implementation of the solution presented in this paper required them to attend training sessions and meetings, as well as review new policies and procedures. Changes in work processes and team dynamics were an additional threat to the implementation of the solution as it was necessary to update team members’ permissions and roles.

Training was another area in which difficulties were encountered as health care providers had to learn new skills and competencies to use the specifications effectively, which involved becoming accustomed to new technology and tools. This was particularly challenging for those who were less comfortable with technology, limiting their willingness to adopt new practices and posing resistance to change.

Finally, it was essential to consider time constraints because the findings discussed in this paper pertain to the duration of the study. The adoption of clinical standards is an ongoing process, and this paper may not be able to fully capture the long-term effects of the implementation.

### Conclusions

Traditional health care is plagued by the use of disparate systems for managing patient data, leading to a fragmented view of medical records as well as inconsistencies and gaps in clinical information. Without standardized and efficient systems in place, there is a higher risk of medical errors, miscommunication, delayed or inadequate diagnoses, and suboptimal treatment decisions, which can ultimately compromise patient safety and health care quality. In addition, this issue underscores the importance of interoperability in health care.

In the case of corneal transplantation, accurate and timely management of patient information is critical for the success of the procedure and the well-being of the patient. Hence, this study proposed the adoption of clinical standards, specifically openEHR, to address these challenges by enabling the creation of a comprehensive and shared patient record. This paper provides insights into the use of openEHR in health care and contributes to the incessant efforts to improve the quality and safety of patient care.

The implementation of openEHR specifications to standardize corneal transplantation records and streamline its workflow can yield significant benefits to patients, health care providers, and the health care system as a whole. Standardized EHRs can ensure the accuracy, consistency, and completeness of data entry and management, leading to increased patient safety and reduced medical errors. Furthermore, it serves as a centralized repository for clinical data, enabling health care providers to access information more easily and facilitating the seamless exchange of data between different health care systems. openEHR can also support clinical decision-making by providing real-time access to patient data and enabling clinicians to make more informed decisions about patient care.

In summary, the process that connects openEHR forms, WPs, and DLMs ensures that the best course of action for a given patient is taken by providing real-time decision support, data validation, and automated actions and ensuring that patient data are accurate, consistent, and accessible, as well as that all necessary clinical tasks are performed in a timely and efficient manner.

Although this study focused on the implementation of clinical standards in a specific health care setting, the principles and strategies used to implement openEHR specifications remain relevant and applicable in other health care contexts. Hence, the findings of this study hold considerable value for health care professionals, hospital administrators, and technology developers, providing critical insights into the implementation of openEHR specifications within a hospital setting and paving the way for the development of innovative solutions to optimize health care operations.

In light of these benefits, it is clear that the adoption of openEHR structures for the standardization of corneal transplantation records represents a critical step forward in the pursuit of safer, more effective, and higher-quality care. Hence, the authors believe that using openEHR specifications will become standard practice in the health care industry in the near future.

Future research could focus on the application of artificial intelligence algorithms to data extracted from standardized EHRs as training algorithms on reliable, consistent, and high-quality data leads to more robust and trustworthy results. This can enable a more efficient and effective clinical data analysis, maximizing the potential of openEHR to drive meaningful improvements in health care outcomes.

## Abbreviations

DL: Decision Language
DLM: Decision Logic Module
EHR: electronic health record
TP: Task Plan
WP: Work Plan
